# Supplementary figures and images for: Value of D-dimer in predicting various clinical outcomes following community-acquired pneumonia: A network meta-analysis
Source: PLoS One. 2022 Feb 23;17(2):e0263215. doi: 10.1371/journal.pone.0263215 (PMC8865637; doi:10.1371/journal.pone.0263215)

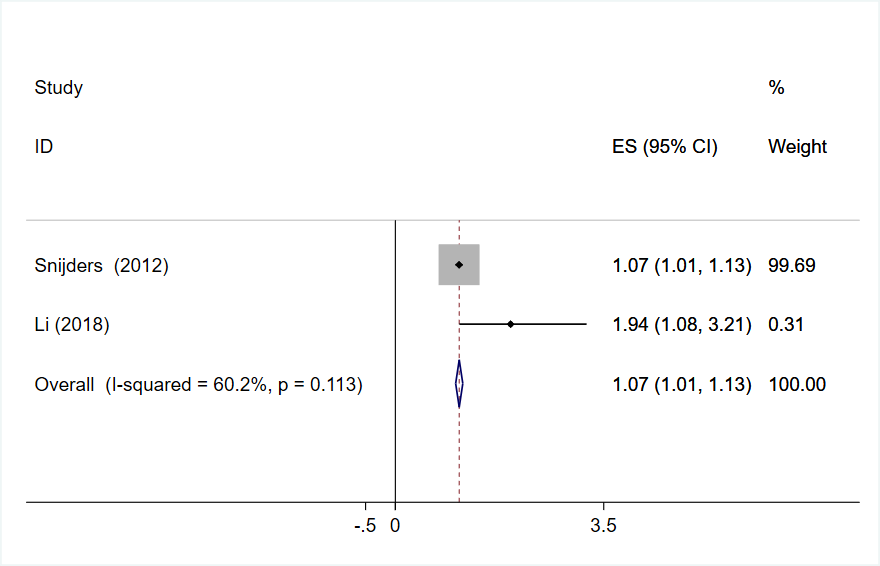

Supplement: S1 Fig — OR, odds ratio. CAP, community-acquired pneumonia. (TIF) [file pone.0263215.s001.tif]

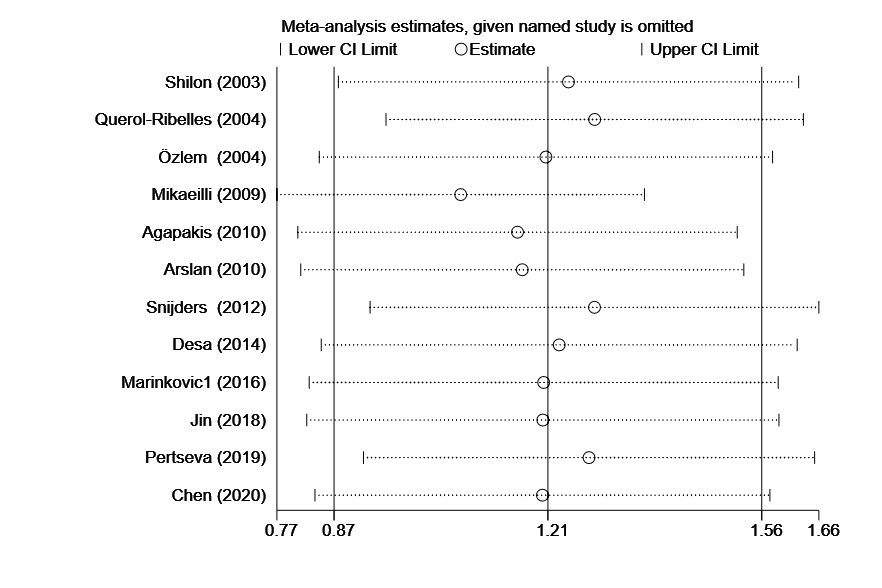

Supplement: S2 Fig — (TIF) [file pone.0263215.s002.tif]

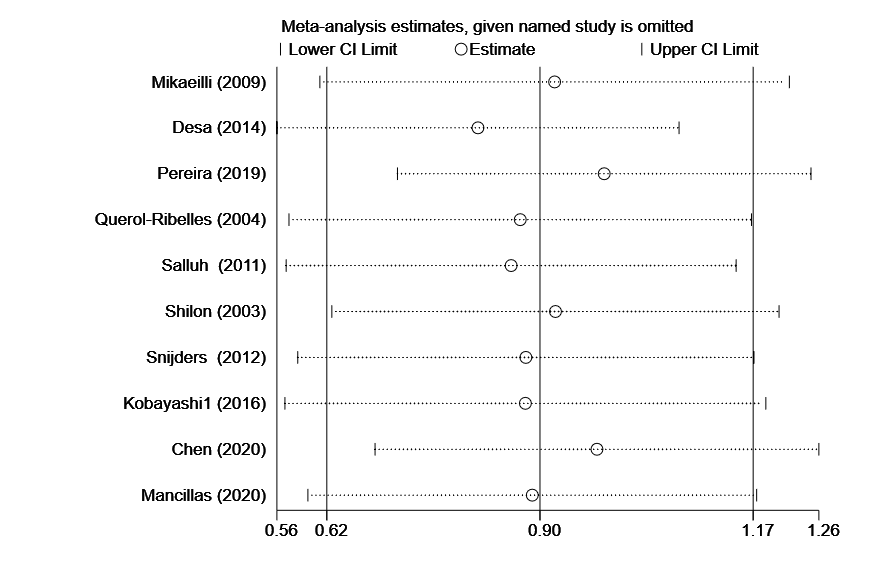

Supplement: S3 Fig — (TIF) [file pone.0263215.s003.tif]

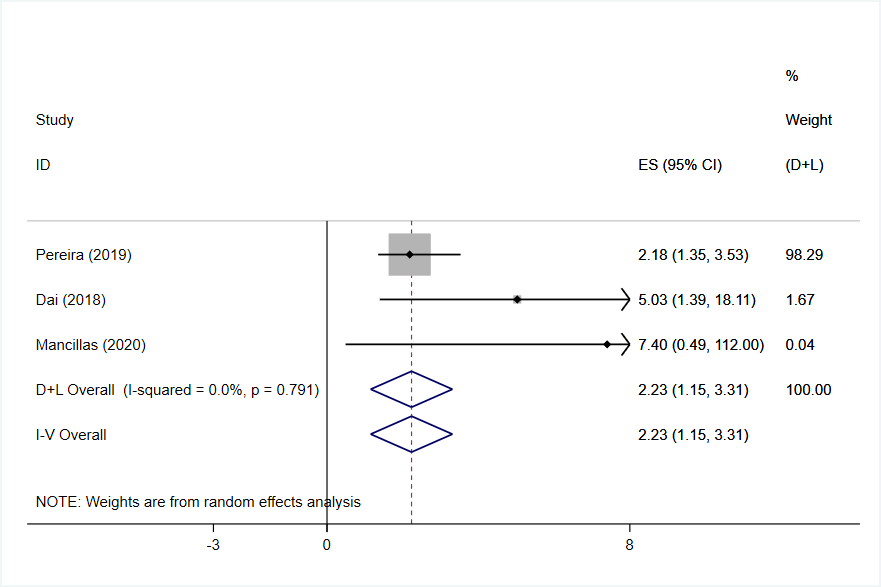

Supplement: S4 Fig — CAP, community-acquired pneumonia. (TIF) [file pone.0263215.s004.tif]

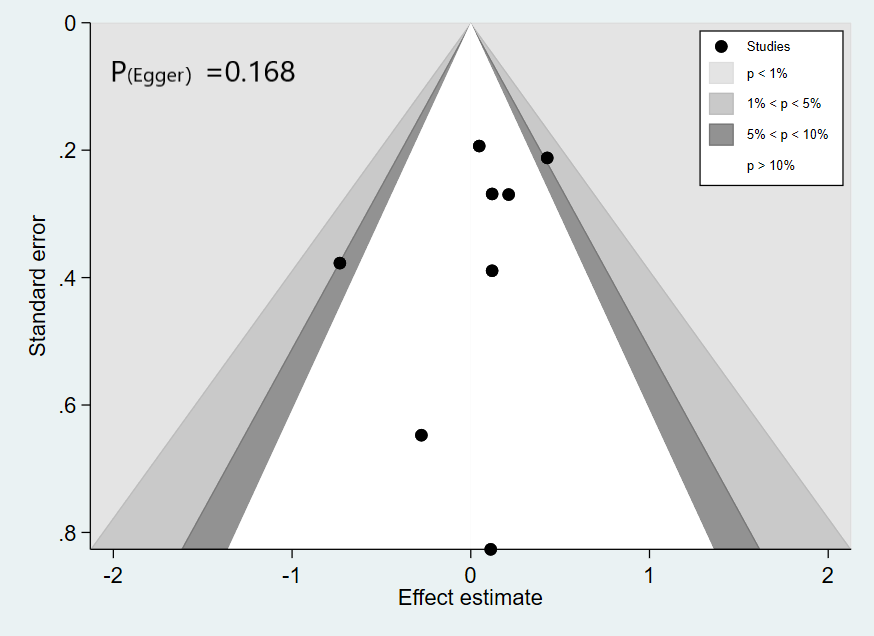

Supplement: S5 Fig — (TIF) [file pone.0263215.s005.tif]

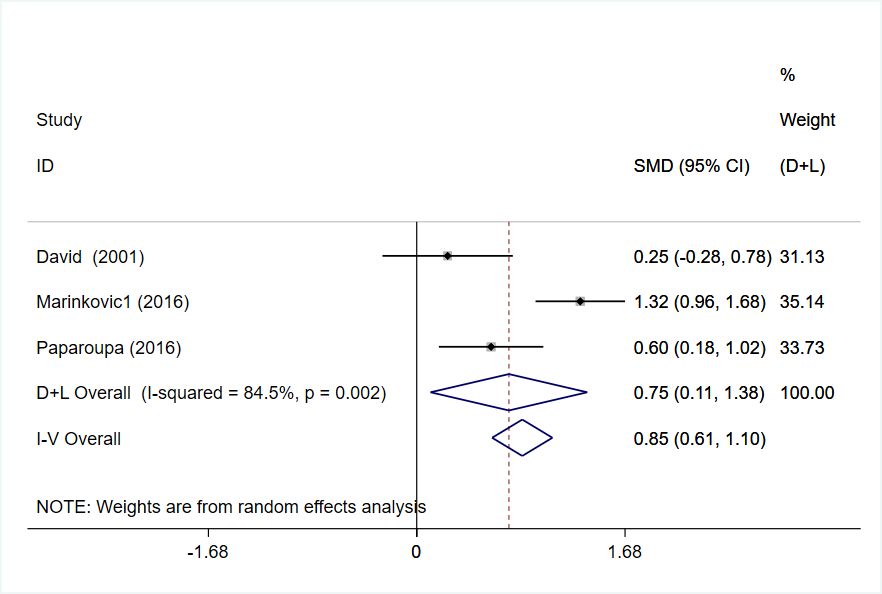

Supplement: S6 Fig — CAP, community-acquired pneumonia. PE, pulmonary embolism. (TIF) [file pone.0263215.s006.tif]

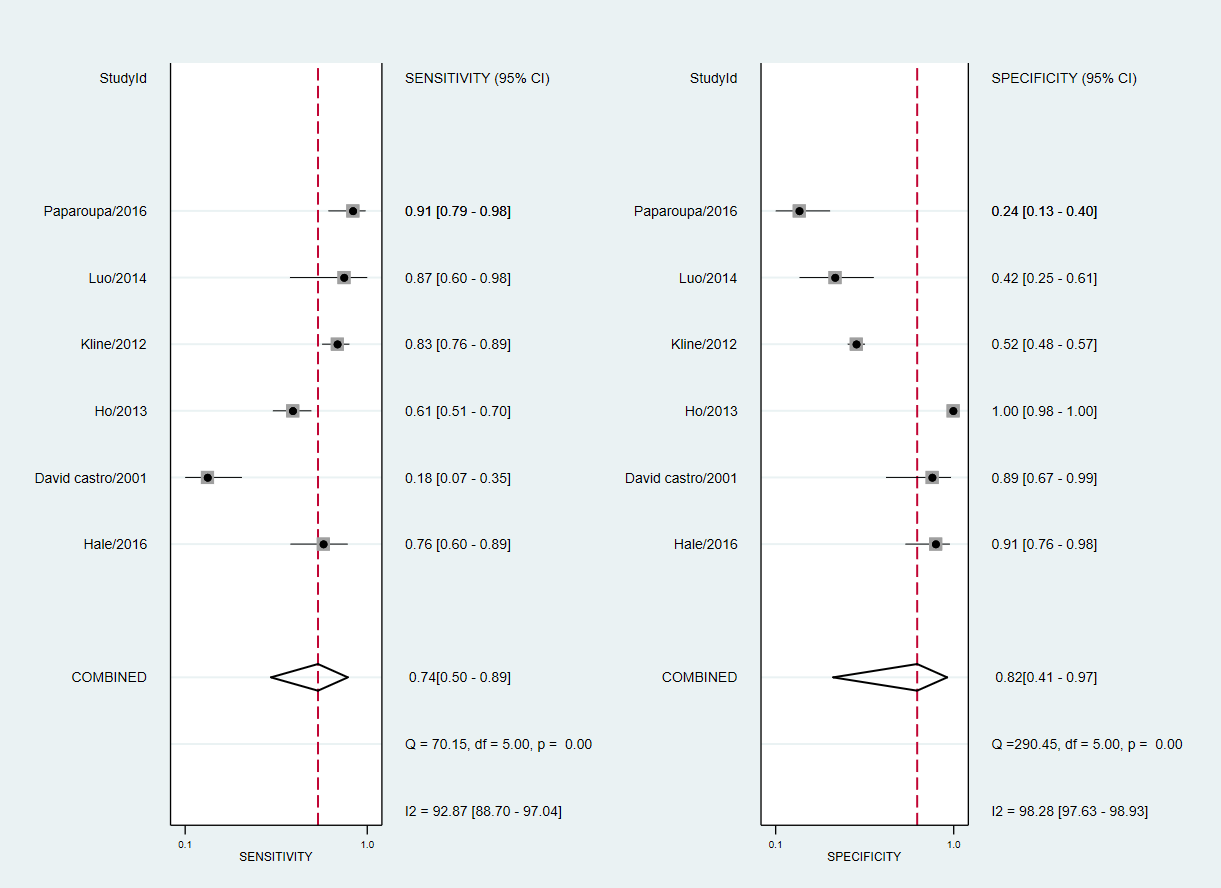

Supplement: S7 Fig — (TIF) [file pone.0263215.s007.tif]

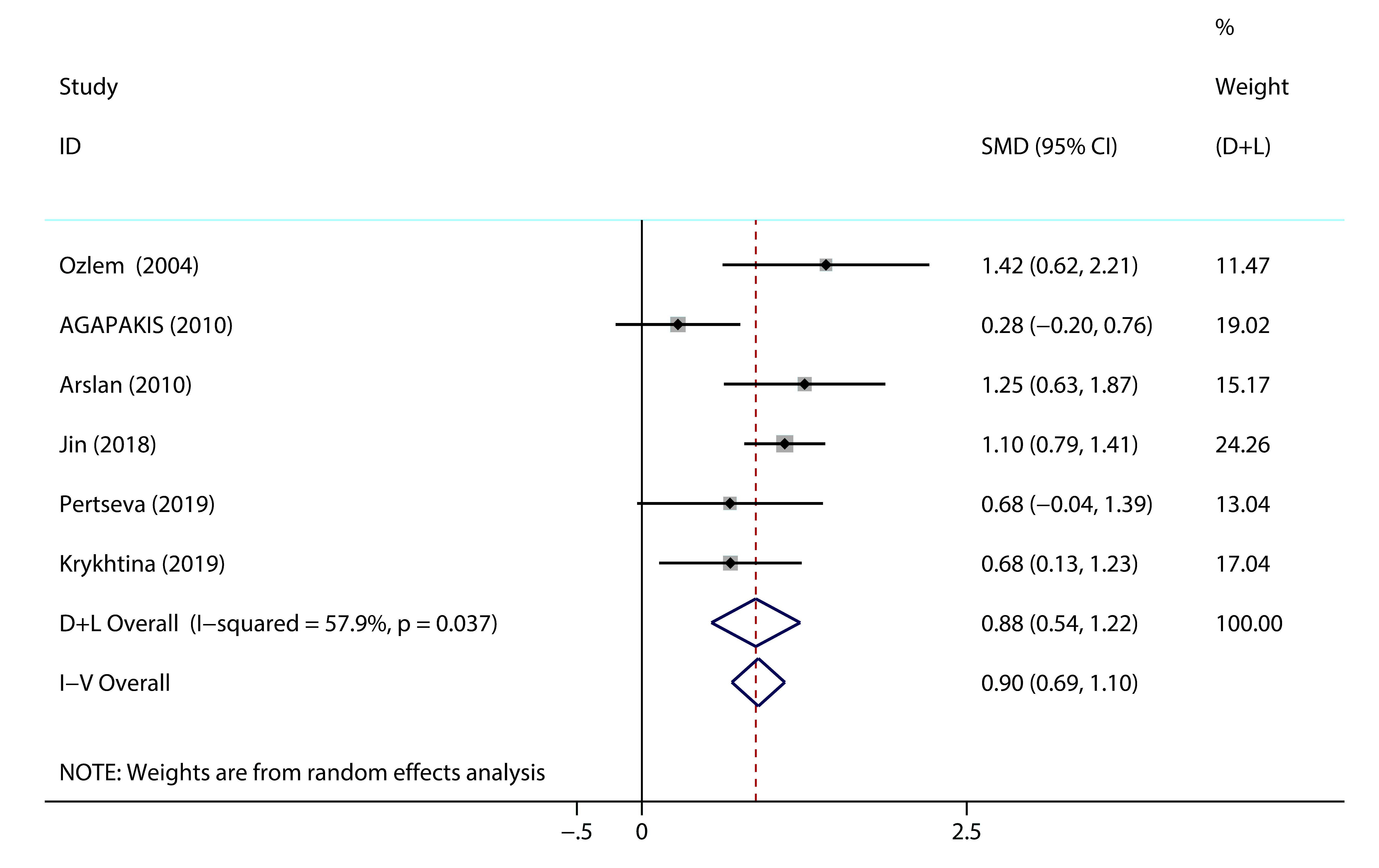

Supplement: S8 Fig — SMD, standardized mean difference; CAP, community-acquired pneumonia. (TIF) [file pone.0263215.s008.tif]

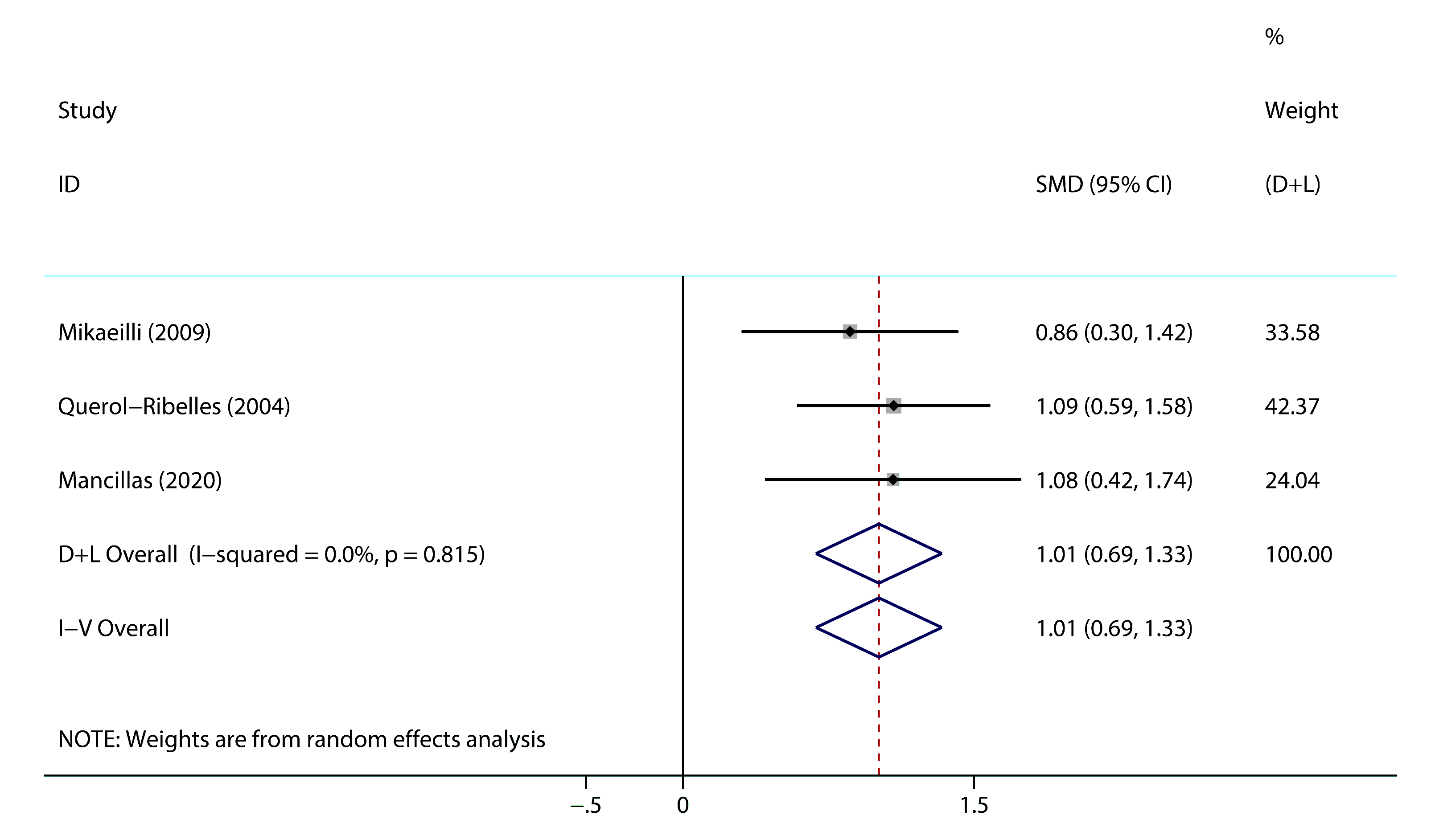

Supplement: S9 Fig — SMD, standardized mean difference; CAP, community-acquired pneumonia. (TIF) [file pone.0263215.s009.tif]

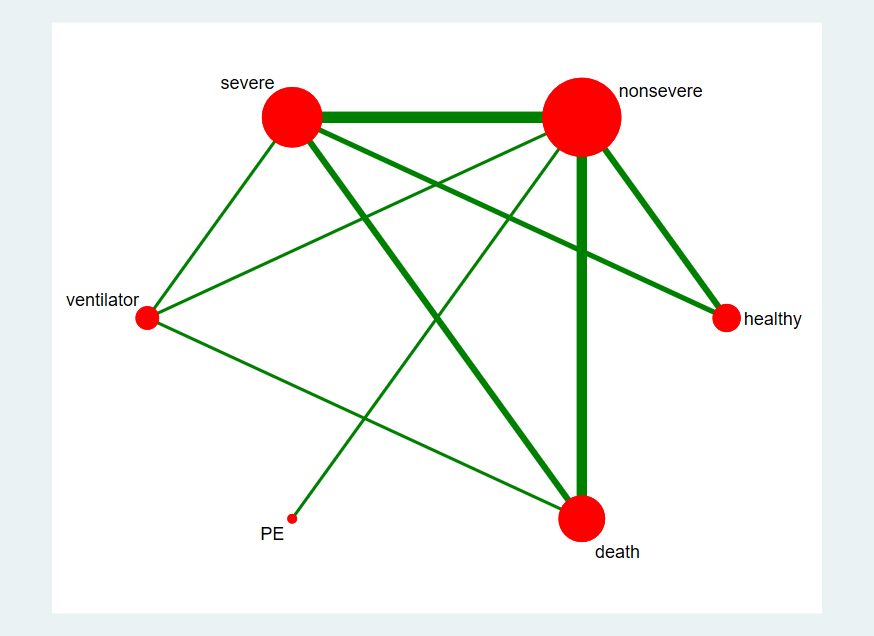

Supplement: S10 Fig — The line width is proportional to the number of trials performed between two outcomes. Circle size is proportional to the total number of patients for each clinical outcome in the network. (TIF) [file pone.0263215.s010.tif]

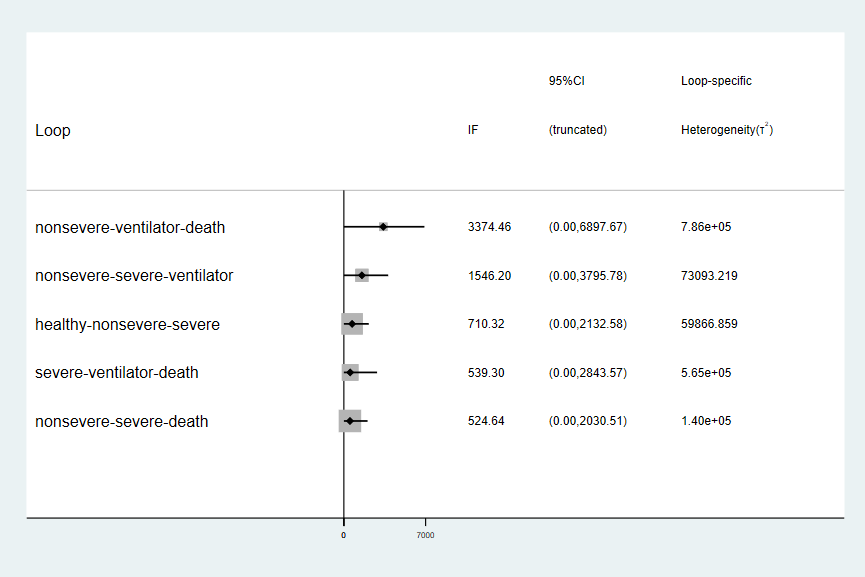

Supplement: S11 Fig — RoR: The rate ratio of logarithms of two ORs of direct and indirect comparisons. (TIF) [file pone.0263215.s011.tif]

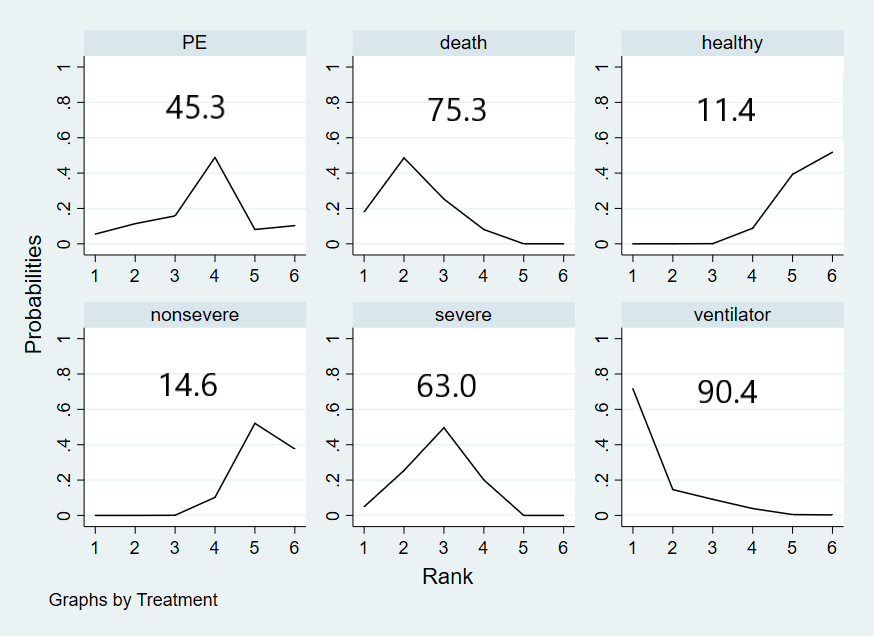

Supplement: S12 Fig — (TIF) [file pone.0263215.s012.tif]

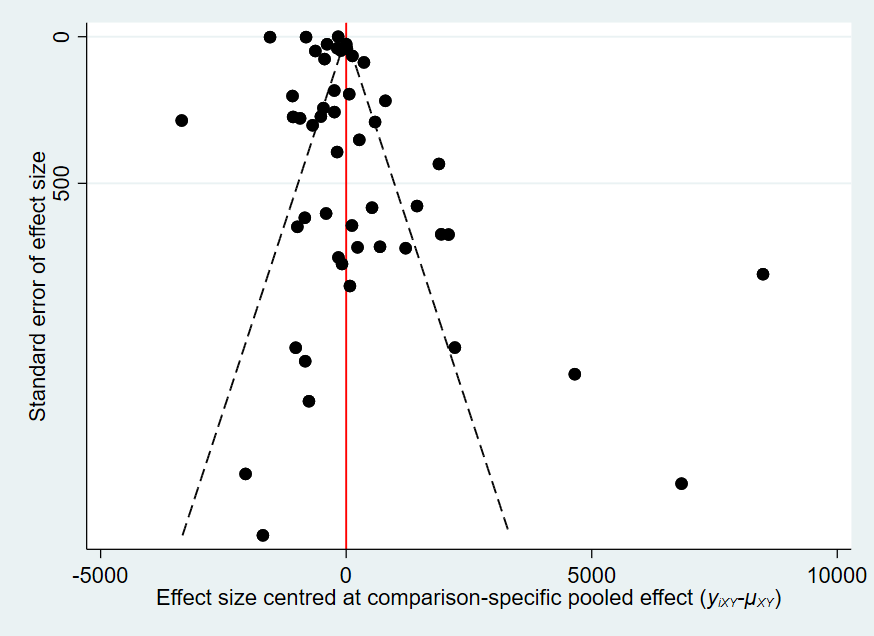

Supplement: S13 Fig — (TIF) [file pone.0263215.s013.tif]
