# Supplementary material for: Value of D-dimer in predicting various clinical outcomes following community-acquired pneumonia: A network meta-analysis
Source: PLoS One. 2022 Feb 23;17(2):e0263215. doi: 10.1371/journal.pone.0263215 (PMC8865637; doi:10.1371/journal.pone.0263215)
Supplement: Attachment — Submitted filename: Response to Reviewers.docx [file pone.0263215.s016.docx]

**Reviewer 1**

1. The authors identified only 1 RCT and the rest are observational study. I don't think it is reasonable to mix results of RCT and observational studies into a meta-analysis. They should be reported, analyzed, discussed, and concluded separately because their data were generated from totally different study designs.

**Response to reviewer:**

We are sorry for this point of confuse. The reference 42 (Snijders, 2012), which was mentioned as one RCT in our article, was a secondary analysis based on a randomized controlled trial. Although the original analysis was in line with principles of RCTs, the secondary analysis based on outcome of patients (for example, d-dimer value of survivor and non-survivors) should be regarded as case-control study design. We have revised its design type as case-control study and examined the robustness of conclusion without this study which was not showed in article. Thanks very much.

2. SMD is standardized mean difference not "standard mean deviation" as it was mentioned in the abbreviation list. This is used for measuring effect of treatment. I am really not sure if this is applicable for prognostic study. It is unclear how the concept of "mean difference" is applied to determine whether d-dimer can be used to predict outcome. I feel it is more straightforward to use OR for this purpose, where a standard cut-off is used to determine high/low d-dimer level.

**Response to reviewer:**

It’s our negligence in abbreviation list where we wrongly used “standard mean deviation” and we have corrected it. As a guide for meta-analysis of prognostic studies published in *BMJ* journal suggested, unadjusted and adjusted prognostic effect estimates (eg, risk ratios, odds ratios, hazard ratios, mean differences) could be results to examine prognostic factors, although adjusted ratios are most recommended. The unadjusted odds ratio which we have transformed to standardized mean difference represented the risk that patients with poor outcome or prognosis had high levels of d-dimers. TAmong included studies for our meta-analysis, only a small proportion of them had supplied adjusted ORs and we have made data synthesis of these ORs. Because no consensus for prognostic meta-analysis, we also examined efficacy of prognostic factors with the help of principle of diagnostic meta-analysis, which regarded patients with poor outcome as positive evens of gold standard and regarded prognostic factors with cutoff as diagnostic method. Dose-response meta-analyses were a relatively ideal method to take full advantage of information and data of primary studies. Unfortunately, undetailed reports of included primary studies made it impossible to make dose-response curves. To be honest, our original intention was to measure the risk ratios that patients with different levels of d-dimer had poor outcome, but the existing studies did not support to complete such a meta-analysis. Therefore, we chose to transform odds ratio to SMD, not vice versa, to directly represent higher d-dimer level in patients with poor outcome. On the other hand, most of included studies reports continuous data and too much transform would bring unpredicted bias. We expected that our work would inspire more researchers to make more scientific studies and report more detailed data. We are trying to make a meta-analysis of individual participant data to solve this problem. Thank you.

3. Variations in methodologies used to measure d-dimer level might have contributed to the significant heterogeneity. The authors have mentioned of doing subgroup analyses. I wonder if variations in methodologies were considered when doing subgroup analyses?

**Response to reviewer:**

We are sorry for absence of mention of this subgroup analysis. We had made subgroup meta-analysis by methodologies of d-dimer measurement, but we didn’t put the figure of synthesis into our article because of too many more important figures. When pooled results showed great heterogeneity, nearly all subgroups including more than 3 studies also have considerable heterogeneity. For example, I-square of subgroups of studies having used Immunoturbidimetric assay, latex immunoassay and ELISA were 86.1%, 96.4% and 96.1%, respectively. We have added this point in our article.

**Reviewer #2:**

1) pg 17 why significant heterogeneity is mentioned as P<0.1, Did this value apply to the whole manuscript?

**Response to reviewer:**

A limitation of Cochran’s Q test is that it might be underpowered when few studies have been included or when event rates are low. Therefore, it is often recommended to adopt a higher [P](https://www.ncbi.nlm.nih.gov/books/n/methresclinhet/abbreviations/def-item/abbreviations.gl1-d106/)-value (rather than 0.05) as a threshold for statistical significance when using Cochran’s Q test to determine statistical heterogeneity. We added relevant reference for this P value in our article.

(2) pg 19

Assessment of methodological quality

QUIPS tool had been used to assess the quality of included studies (Table 2). Among all the 32 studies, 20 were high risk of bias [11, 30, 31, 33, 34, 36-41, 43, 48, 53-55, 57-60] and only 2 studies were evaluated as low risk of bias [42, 52]. The number of individual domains rated as high risk ranged from 0 to 3.

Is there any specific reason that why moderate category is excluded from the discussing in the “Data Extraction and Study Quality Assessment component as well as from the “results” session.

**Response to reviewer:**

We are sorry for our confusing description. What we wanted to make readers knew was that 20 included studies were high risk, 2 were low risk and the remaining 10 studies were medium risk of bias. We have revised it. Thank you.

3) Results session

- kindly recheck the following data:

o pooled result of figure 4 SMD=1.22 95%CI 0.67-1.77, I2=85.1% p=0.000 written in the paragraph and the data from the figure

o supplementary figure 4 – 3 studies or 4 studies, pooled result

"Pooled results of adjusted ORs from 4 studies [50-52, 54] have confirmed the prognostic value of increased D dimer (ES=0.90 95%CI 0.62-1.17, I2=59.4% p=0.008) (Supplementary Figure. 4). We didn’t find factors contributing to the heterogeneity by subgroup analysis"

Kindly recheck which was written in the paragraph and the data from the figure

o pooled result of supplementary figure 6

Pooled effect based on continuous data from three studies suggest a significant elevation in CAP patients with PE than those without PE (ES=1.07 95%CI 1.01-1.13, I2=60.2% p=0.113). (Supplementary Figure. 6).

kindly recheck which was written in the paragraph and the data from the figure

o some data of supplementary figure 7

I2=81.4%

kindly recheck which was written in the paragraph and the data from the figure

- for the “others” kindly refer with the respective figures

Pooled effects suggested patients with CAP had a higher level of d-dimer compared to healthy participants (SMD=0.88, 95%CI 0.54-1.22, I2=57.9 %, p=0.037) and D-dimer was significantly further elevated in patients requiring invasive mechanical ventilator (SMD=1.01, 95%CI 0.69-1.33, I2=0.0%, p=0.815).

(Kindly add "Referencing figures")

**Response to reviewer:**

We are sorry for our serious negligence to mistakenly correspond valued of pooled results to figures. Thanks for your correction with patience and we have revised our article according to your suggestion. Thank you very much.

4) page 22

- 2nd paragraph – “this is may be” – incorrect use, please update

**Response to reviewer:**

We have modified it to “this is partly because of …”. Thanks for your advice.

5) I would suggest to avoid using “it’s” “didn’t” – instead kindly use "it is or did not" and some minor punctuation and grammar usage.

**Response to reviewer:**

We have replace with “it is or did not” in our revised article. Thank you.
